# The utility of dynamic contrast-enhanced intranodal magnetic resonance lymphangiography (MRL) in the investigation of primary lymphatic anomalies

**DOI:** 10.1016/j.crad.2024.06.009

**Published:** 2024-10

**Authors:** L.A. Ratnam, M. Mills, A. Wale, L.R. Howroyd, M. Itkin, F.A. Howe, K. Gordon, S. Mansour, P. Ostergaard, P.S. Mortimer

**Affiliations:** a Department of Radiology, St George's University Hospitals NHS Foundation Trust, London SW17 0QT, UK; b Molecular and Clinical Sciences Research Institute, St George's University of London, London SW17 0RE, UK; c Dermatology and Lymphovascular Medicine, St George's University Hospitals NHS Foundation Trust, London SW17 0QT, UK; d South West Thames Centre for Genomics, St George's University Hospitals NHS Foundation Trust, London, UK; e Department of Radiology, University of Pennsylvania Health System, Philadelphia, USA

## Abstract

**AIM:**

The aim of this study was to describe the technique of DCMRL to identify central lymphatic abnormalities in patients with primary lymphatic anomalies and discuss utility of the findings.

**MATERIALS AND METHODS:**

Twenty-eight patients with primary lymphatic abnormalities underwent dynamic magnetic resonance imaging (MRI) following injection of gadolinium directly into inguinal lymph nodes at a tertiary lymphovascular referral center.

**RESULTS:**

Technical success was achieved in 23 patients (82.1%). Pathological imaging findings included obstructed, hypoplastic, or absent lymphatic channels with collateralization/rerouting or reflux of flow, lymphangiectasia, lymphatic pseudoaneurysms, and lymph leaks. Protocol modifications for improved imaging are highlighted including technical aspects of lymph node injection, image acquisition and MRI parameters. In two patients, imaging findings warranted embolization of the abnormal lymphatic channels with subsequent symptomatic improvement.

**CONCLUSION:**

DCMRL has been shown to be a safe, reproducible technique in patients with primary lymphatic anomalies enabling imaging of the central lymphatic system.

## Highlights

• Central lymphatics visualized in 82.1% with dynamic contrast-enhanced intranodal magnetic resonance lymphangiography (DCMRL).
• Optimizing imaging parameters and needle placement technique are critical.
• DCMRL enables dynamic assessment of central lymphatics with anatomical correlation.

## Abbreviations

CNR: Contrast-to-noise ratio
DCMRL: Dynamic contrast-enhanced intranodal magnetic resonance lymphangiography
MIP: Maximum-intensity projection
MR: Magnetic resonance
MRA: Magnetic resonance angiography
MRL: Magnetic resonance lymphangiography
SCID: Severe combined immunodeficiency
SPGR: Spoiled gradient echo
TD: Thoracic duct

## Introduction

Intranodal dynamic contrast enhanced magnetic resonance lymphangiography (DCMRL) is a relatively new technique enabling imaging of the central lymphatics via injection of contrast directly into inguinal lymph nodes. Lymphoscintigraphy remains the investigation of choice for diagnosis in lymphatic disorders but does not give detailed imaging of the central lymphatics.^1^, ^2^, ^3^ Pedal lymphangiography with Lipiodol whereby contrast was injected directly into lymphatics in the foot which had been surgically exposed is invasive, very time consuming, involves ionizing radiation, and can result in lipiodol embolization.^4^ St Georges University Hospitals NHS Foundation Trust is recognized as a national referral centre in the UK for Primary Lymphatic Anomalies. Many of these genetically determined forms of primary lymphatic anomalies have central lymphatic abnormalities associated with (or causing) peripheral lymphedema.^5^, ^6^, ^7^, ^8^ These lymphatic abnormalities may not be clinically obvious yet are important to diagnose both for correct phenotyping and for ongoing management.^9^ Although described for other indications, there remains a paucity of data exploring DCMRL's utility in patients with primary lymphatic anomalies.^10^, ^11^, ^12^ The purpose of this paper is to describe the method and utility of this technique in imaging central lymphatic abnormalities in patients with primary lymphatic anomalies.

## Materials and methods

### Patients

Patients were referred for DCMRL if they had a diagnosis of a primary lymphatic anomaly (chylous reflux, significant lymphedema with a suggestion of obstruction on lymphoscintigraphy, chylothorax, chylopericardium, or chylous ascites).

### DCMRL technique

Inguinal lymph node puncture was carried out under ultrasound guidance using a shallow angle of entry with a long subcutaneous tract to obtain a stable needle position with a 23G spinal needle (BD, Franklin Lakes, NJ). Ideally, a minimum of one needle was sited in each groin. Where nodes were easily visualized and patients tolerated placement well, a third needle was sited to increase likelihood of contrast uptake. Satisfactory position within the lymph node was then confirmed using injection of 1 ml of SonoVue (*sulfur hexafluoride microbubbles)* ultrasound contrast (Bracco Spa, Milan, Italy) mixed with 2 ml of 0.25% Chirocaine (levobupivacaine) for local anesthesia. The needles were then secured in place with dressings and connecting tubing attached with syringes primed with gadoteric acid (Dotarem®; Guerbet, France).

### Magnetic resonance imaging

For all studies, magnetic resonance (MR) imaging was performed using a clinical 3.0-T magnetic resonance imaging (MRI) system, Philips Achieva 3.0T TX, and a 16-element phased array torso coil for signal reception. The imaging protocol (Table 1) consists of initial non-contrast sequences followed by dynamic contrast enhanced imaging.

**Table 1:** Typical imaging parameters for magnetic resonance lymphangiography at 3.0 T. Note that field of view and acquisition matrix varied from participant to participant based on the anatomy. Image acquisition times varied based on the field of view required for adequate anatomical coverage but were generally between 6 and 10 minutes for the heavily T_2_-weighted and 0.5–1 minutes per volume for the dynamic T_1_-weighted series.

	TR/TE (ms)	FA (^0^)	Reconstructed voxel size (mm)	NSA	Fat suppression	Motion reduction	SENSE factor (direction)	Typical acquisition time	Features of interest
Precontrast:									
Coronal 2D T_2_-weighted TSE	‘shortest'^a^/80 ms	90	0.78 × 0.78 × 4.00	1	SPAIR	Breath-held	2.0 (RL)	5 min	Fluid accumulations (e.g., ascites), anatomy, incidental findings
Coronal 3D heavily T_2_-weighted TSE	3200/740	90	0.90 × 0.90 × 1.50	2	SPAIR	N/A	1.6 (RL) 1.6 (AP)	9 min	Fluid accumulations, occasionally LV
Coronal 3D T_1_-weighted Spoiled gradient echo	‘shortest’/‘shortest’^a^	30	0.76 × 0.76 × 1.50	1	N/A	N/A	3.0 (RL)	0.5 min	Baseline prior to contrast injection
Post-contrast:									
Coronal 3D T_1_-weighted Spoiled gradient echo	‘shortest’/‘shortest’^a^	30	0.76 × 0.76 × 1.50	1	N/A	N/A	3.0 (RL)	0.5 min/dynamic	Enhancing LV, and contrast leakage/pooling
Coronal 3D T_1_-weighted Dixon	4.36/1.41/2.60^b^	10	0.71 × 0.71 × 1.00	1	Dixon	N/A	1.6 (RL) 1.6 (AP)	2 min	Enhancing LV, and contrast leakage/pooling

TR: repetition time, TE: echo time, FA: flip angle, NSA: number of signal averages, SENSE factor: SENSitivity Encoding.

RL: right–left; AP: anterior–pPosterior; LV: lymphatic vessels; TSE: turbo spin echo; 2D: two-dimensional; 3D: three-dimensional.

a 'shortest’ TR for the Coronal 2D T_2_-weighted TSE was approx. 2000ms; ‘shortest’ TR/TE for the Coronal 3D T_1_-weighted spoiled gradient echo were approx. 5/2 ms.

b TE_1_ = 1.41 ms, TE_2_ = 2.60 ms.

Noncontrast imaging of the abdomen and pelvis begins with a two-dimensional (2D) T_2_-weighted breath-held turbo spin echo (TSE) acquisition for identification of gross abnormalities (e.g., fluid accumulations), three-dimensional (3D) image planning and allows for the identification of incidental findings. A 3D heavily T_2_-weighted TSE sequence follows, for which only very long T_2_ compartments retain reasonable signal intensity. Finally, a precontrast 3D T_1_-weighted spoiled gradient echo (SPGR) image was acquired. The non-contrast T2-weighted images were assessed for the presence or absence of ascites, pleural, and pericardial effusions, edema in the soft tissues and the presence of masses of lymphatic nature, and for how well the lymphatics were visualized.

Slow injection (over 1–2 minutes) of 4–9ml of undiluted 279.32 mg/ml gadoteric acid (Dotarem®; Guerbet, France) was then carried out simultaneously via each needle placed in an inguinal lymph node, followed by dynamic T_1_-weighted imaging post injection to depict contrast dispersion over time (each acquisition lasted 0.5 minutes). A maximum of 3 needles were placed and a maximum total dose of 18 ml of gadoteric acid was injected. In cases where contrast was initially difficult to visualize, T_1_-weighted Dixon images, of higher spatial resolution and larger field of view than the spoiled gradient echo sequences, were acquired. Imaging initially focused on the pelvis and lower abdomen until contrast was seen to leave this region (variable), at which point, the coil was repositioned to continue imaging to the thoracic duct (TD) termination. The postcontrast T_1_-weighted images were evaluated for presence or absence of lymphatic vessels, anatomical distribution of the contrast (normal or abnormal), reflux, lymphatic pseudoaneurysms, leakage of lymphatic fluid, rerouting/collateralisation, and the presence of dermal backflow of contrast. All image volumes were reformatted as maximum-intensity projections (MIPs) for review, coronal projections for T_1_-weighted image series, and radial projections for the T_2_-weighted images. Total imaging time ranged from approx. 30–90 minutes; however, an hour was typical (average ± standard deviation = 53 ± 13 min), and variability was secondary to individual variation in the postcontrast acquisition time.

## Results

From January 2018 to December 2022, 28 patients were imaged. A summary of the results and underlying primary lymphatic diagnosis is provided in Table 2. The imaging findings are described in the following. No contrast reactions were observed, and no patients terminated the examination due to pain, no delayed complications were reported during post procedure follow-up.

**Table 2:** Summary of patients and DCMRL findings.

Patient No	Sex	Age at time of study (years)	Lymphatic diagnosis	Unilateral injection	Bilateral injection with no uptake	Bilateral injection with bilateral uptake	Bilateral injection with unilateral uptake
1	M	18	WILD syndrome^22^		x		
2	M	38	WILD syndrome		x		
3	M	31	RASopathy (Noonan syndrome)			TD terminates at ligation.	
4	F	39	RASopathy (Noonan syndrome)				Mediastinal, pleural & pericardial leak.
5	F	50	YNS			Normal central lymphatics.	
6	M	61	YNS			Absent TD, collateral filling, LPSA.	
7	F	47	GLD			Dilated lymphatic vessels.	
8	F	25	GLD			Absent TD, collateral filling.	
9	M	46	GLD		x		
10	M	19	GLD	CL severe edema. CL contrast- reflux.			
11	F	70	GLD			Obstruction with rerouting and filling of distal TD.	
12	M	31	GLD				LPSA, absent lower TD (ligated). Distal filling via collaterals.
13	M	42	GLD	CL severe edema. Superficial rerouting.			
14	M	24	*ERG*-related GLD^23^				Obstruction with rerouting to TD.
15	F	41	*ERG*-related GLD		x		
16	M	29	SCIDS			Normal central lymphatics.	
17	M	38	Unilateral leg lymphedema (R)		x		
18	F	16	Unilateral leg lymphedema (L)				Absent TD, collateral filling.
19	F	30	Unilateral leg lymphedema (R)			Normal central lymphatics.	
20	F	39	Unilateral leg lymphedema (L)	CL severe edema. Normal on side of uptake and centrally.			
21	F	25	Unilateral leg lymphedema (L)				Normal on side of uptake and centrally.
22	M	29	Left perineal lymphovascular malformation				Normal on side of uptake and centrally.
23	M	33	Left hindquarter lymphovascular malformation, chylous reflux, lymph leakage				Ipsilateral reflux to pelvis and leg.
24	F	52	Bilateral lower limb and abdominal wall lymphedema, chylous ascites & pleural effusions.				LPSA, dilated TD with distal TD obstruction.
25	M	34	Unilateral leg lymphedema (L), chylous ascites & pleural effusions			Absent distal TD, LPSA.	
26	M	62	Genital and right leg lymphedema, chylous reflux, lymph leakage	CL surgery. CL contrast- reflux.			
27	M	17	Genital and bilateral lower limb lymphedema				Obstruction with rerouting to CC.
28	M	64	Genital and right leg lymphedema, chylous reflux, lymph leakage	CL surgery. CL contrast- reflux.			

Abbreviation Key: CC: cisterna chylii; CL: contralateral; GLD: generalized Lymphatic Dysplasia; L: left; LPSA lymphopseudoaneurysm; R: right; SCIDS: severe combined immunodeficiency syndrome; TD: thoracic duct; WILD: warts, immunodeficiency, lymphedema, and anogenital dysplasia; YNS: yellow nail syndrome.

### Noncontrast

**Figure 1:**
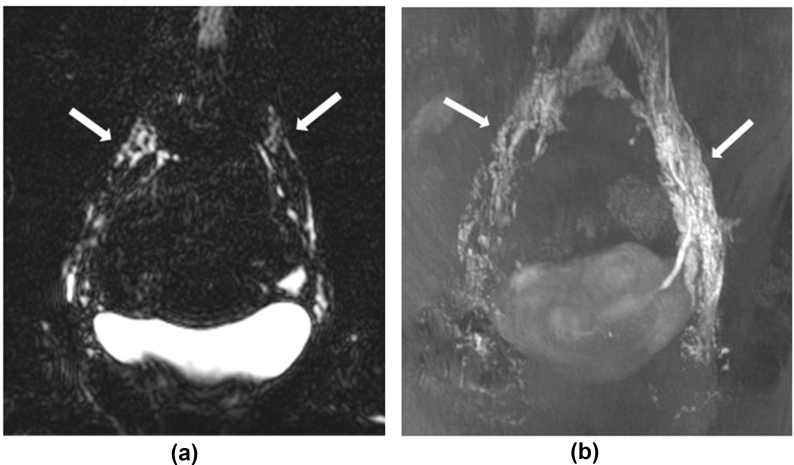
Comparison of non contrast and DCMRL images of the iliac and lumbar lymphatics in a 30-year-old female patient (Case 19) with unilateral right leg lymphedema. Images shown are over the abdomen and pelvis from the lower lumbar vertebrae to the level of the femoral heads. The lymphatics (white arrows) are faintly visualized on the T_2_-weighted noncontrast imaging, with a central slice of the 3D acquisition shown here (1a) and seen much more clearly on the postcontrast T_1_-weighted imaging, as demonstrated in this MIP SPOILED GRADIENT ECHO image (1b). DCMRL showed normal central lymphatics with good bilateral drainage. Abbreviations: DCMRL = dynamic contrast-enhanced intranodal magnetic resonance lymphangiography; MIP = maximum-intensity projection; 3D = three-dimensional.

**Figure 2:**
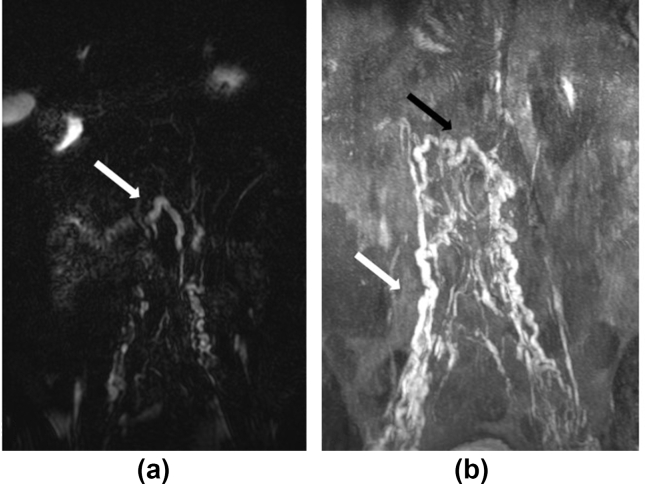
**One of two patients in whom lymphatics were visualized on noncontrast imaging is a** 64-year-old male patient (Case 28) with chylous reflux into right thigh and genitalia and right thigh and genital lymphedema. The first image encompasses the lower thorax to the level of the femoral heads. Markedly dilated lymphatics (white arrow) are reasonably well seen on the noncontrast T_2_ images, as can be seen in the single central slice of the 3D acquisition shown (2a) **The same dilated lymphatic channel is shown in the post contrast image (black arrow) .** Contrast was only injected from left groin as nodes had been surgically removed on the right. However, significant reflux into the right side was identified (white arrow) on post–contrast T_1_-weighted SPRG image, displayed in MIP form here (2b). Dynamic imaging demonstrated the opacification of the right-sided channels after injection of contrast from the left. Abbreviations: SPGR = spoiled gradient echo; MIP = maximum-intensity projection; 3D = three-dimensional.

From the T_2_ noncontrast imaging, central lymphatic channels could not be identified in 10 of the 28 patients. Of the remaining 18 patients, the lymphatic channels could be seen but were faint and not deemed to be sufficiently visualized to be diagnostically helpful in 16 patients [Fig 1]. The lymphatic channels were seen well in only two patients [Fig 2a].

### Post contrast

Technical success visualizing the central lymphatics was achieved in 23 patients (82.1%). In five patients, injection was performed unilaterally due to absence/inability to identify targetable lymph nodes on the contralateral groin either due to previous surgical removal of nodes or du to extensive edema [Table 2]. Central lymphatics were successfully visualized in all five patients, with contrast noted on the noninjected side signifying reflux (cases 10, 26, and 28) [Fig 2b], normal unilateral uptake with normal TD (Case 20), and superficial rerouting indicating obstructed main drainage routes (Case 13).

In the remaining 23 patients, bilateral injections were performed. Of these,

a) in 5 patients (cases 1, 2, 9, 15, and 17), no propagation of contrast was seen bilaterally. These were considered technical failures.
b) in 3 patients (cases 5, 16, and 19), bilateral uptake of contrast was noted, and the imaging showed normal central lymphatics.
c) in 6 patients (cases 3, 6, 7, 8, 11, and 25), bilateral uptake of contrast was noted with abnormalities detected (termination of the TD, absent filling of TD, lymphopseudoaneurysms, lymphangiectasia, and obstructed or absent lymphatic channels with collateralisation).
d) in 9 patients, propagation of contrast was absent on **one** of the injected sides in the groin (bilateral injections performed). Of these, 2 patients (cases 21 and 22) showed normal lumbar and iliac lymphatics on the side with contrast propagation with normal appearances of the TD. The remaining 7 had a range of abnormalities including reflux into the ipsilateral pelvis and limb which was the limb affected with lymphedema (Case 23), collateralisation with re-routing of the lymphatic fluid to the distal lymphatic channels (Cases 14, 18 and 27) and lymphopseudoaneurysms (cases 12 and 24) and a patient with Noonan syndrome with abnormal mediastinal and pulmonary lymphatic perfusion and rapid flow of contrast to the terminal TD (Case 4).

## Discussion

The ability to image the central lymphatic system in this group of patients has led to an understanding of the anatomy of their central lymphatics, which was previously unknown. The discussion is divided into technical factors and the lymphatic findings.

### Review of DCMRL procedures

#### Selection of lymph node for contrast injection

Subjectively abnormal, absent and hypoplastic lymph nodes are more common in patients with primary lymphatic disorders with target nodes frequently measuring <1 cm in size. Ideal needle-tip positioning is at the corticomedullary junction. Lymph node enhancement and efferent flow after injection of ultrasound contrast confirms good positioning.^13^, ^14^, ^15^ The chirocaine alleviates pain that is otherwise experienced on injection of gadoteric acid intranodally.

#### Securing the needles in position

We noted the ease of needle displacement in our initial studies (loss of position between placement and fixation, extravasation noted in the groin) with no uptake of contrast on the displaced side. Thus, if patients tolerated needle placement well, up to 3 needles in one groin were placed to maximize chances of introducing contrast into lymph nodes. This was only carried out in 3 patients. Initial studies had an attempted injection of 9 ml of gadoteric acid into each node; however, reduction to 4 ml injected into each node was found to still provide satisfactory contrast visualization, with improved tolerance from patients. We found that avoiding placement of needles in the groin crease, positioning the coil above the tip of the needles, and the use of connecting tubing primed with contrast and attached at needle placement also stops the needles from becoming displaced when connecting a syringe to the needle for injection in the MRI scanner.

#### Non-contrast T_2_-weighted magnetic resonance imaging

**Figure 3:**
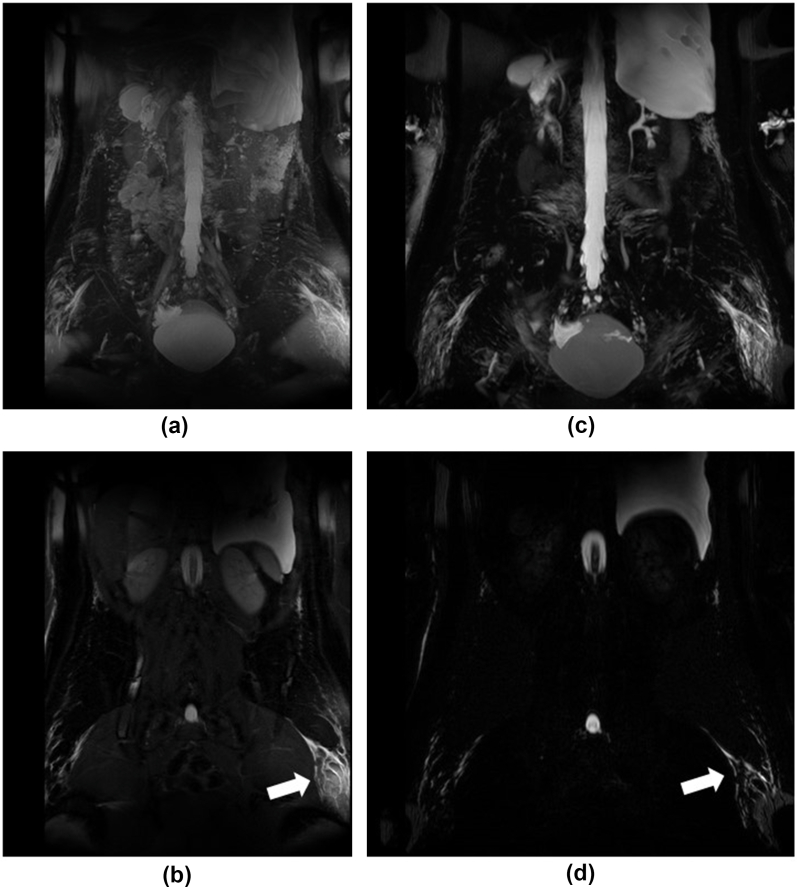
T_2_-weighted images of a patient (25-year-old female, Case 8) with enlarged inguinal lymphatic vessels. (3a) shows the MIP resulting from the 2D T_2_-weighted TSE sequences, whereas (3b) is a mid-image slice from this sequence. (3c) and (3d) are the equivalent from the 3D heavily T_2_-weighted sequence. Note that while more of the anatomy is visible in a–b, the reduced background signal improves lymphatic vessels and fluid pooling visibility with the 3D acquisition (c–d). Note too that visualization of lymphatic vessels is rarer in the more moderately 2D T_2_-weighted images (a–b) than the 3D (c–d). The arrows highlight lymphatics in the upper thigh on the patient's left. Abbreviations: MIP = maximum-intensity projection; 3D = three-dimensional; 2D = two-dimensional; TSE = turbo spin echo.

Unlike most bodily tissues, the lymph can be expected to retain reasonable signal in long-echo-time scans, given its T_2_ time of approx. 610 msec at 3T16. These images provide a high contrast-to-noise ratio (CNR) for fluid-containing regions and are thus especially useful for identifying areas of lymphatic fluid accumulation [Fig 3]. Despite a reduced CNR, moderately T_2_-weighted 2D sequences were also found to be beneficial as the fluid accumulations could be observed in the context of the underlying anatomy. Sites setting up their own MRL protocols may wish to consider whether two acquisitions are required in their context, particularly if scanner time is limited. Acceleration techniques such as partial Fourier reconstruction can also be applied to reduce imaging time but can reduce image SNR and introduce artefacts.^17^

Other noncontrast approaches have been described to assess the lymphatics in the abdomen including a recent attempt using balanced steady state free-process (bSSFP).^18^ In this paper, cardiac triggered and respiratory navigated bSSFP images facilitated improved visualization of the lymphatics compared to respiratory triggered heavily T_2_-weighted TSE. bSSFP sequences exhibit a complex T_2_/T_1_ weighting in which fluids (lymph, CSF, blood) are high signal. We therefore chose not to use bSSFP over TSE, given the t-high blood signal, which could cause confusion in differentiating lymphatic from the vascular structures.

#### Contrast-enhanced T_1_-weighted magnetic resonance imaging

Administration of gadoteric acid into the lymphatics reduces the long T_1_ time of lymph sufficiently to be observed in dynamic T_1_-weighted images where the passage of contrast agent within the lymphatics needs to be observed over time [Fig 3].^16^ We used gadoteric acid due to a combination of local availability and its strong safety profile.^19^ Similar enhancement with a reduced volume of contrast agent may be possible using a similarly safe agent with greater r_1_ relaxivity such as Gadoteridol or Gadobutrol ^20^ but requires *in-vivo* validation for lymphatic applications.

**Figure 4:**
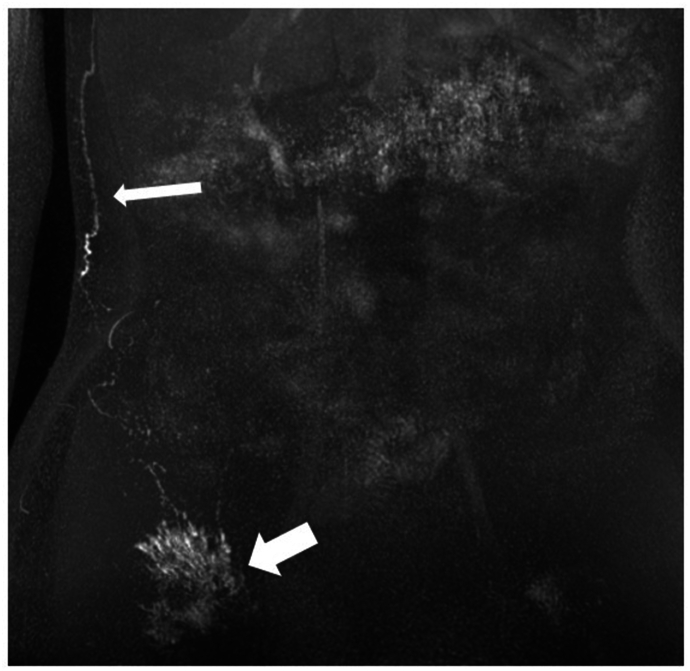
Postcontrast T_1_-weighted Dixon maximum-intensity projection (MIP) of a 24-year-old male patient (Case 14) with no drainage into normal lymphatic vessels showing superficial rerouting of contrast injected into a right groin lymph node (thick white arrow shows contrast partially extravasated around the right groin lymph node) up the right flank (thin white arrow showing contrast re-routing along right flank).

With high spatial and temporal resolution [Table 1], the spoiled gradient echo sequence was the default sequence for our dynamic imaging, Dixon-based images are less affected by inhomogeneities outside the scanner isocentre but maintain high quality fat suppression, DIXON imaging was acquired in several cases and was particularly useful for cases in which the contrast agent was seen to reroute via superficial lymphatic vessels [Fig 4]. A spoiled-gradient echo sequence was chosen as the standard sequence for the dynamic post–contrast imaging as DIXON imaging takes longer to reconstruct and therefore limits the ability for on-table evaluation of the findings.

Some sites performing DCMRL have used keyhole imaging to accelerate their T_1_-weighted dynamic imaging.^21^ However, care must be taken to ensure sufficient high spatial frequency data are captured to appropriately reconstruct small structures such as lymphatics and further work in this area is required. Additionally, methods of motion artefact reduction have been used by several centers (breath-held T_1_-weighted contrast enhanced scans and respiratory or cardiac gating/triggering T_2_ scans) to reduce motion artefacts and improve image quality at the expense of extend imaging times and can be considered if time is not a limiting factor.^22^, ^23^, ^24^, ^25^, ^26^, ^27^, ^28^, ^29^

### Observation of lymphatic abnormalities

#### Absence of contrast uptake

Five patients with absent bilateral contrast uptake were also found to have no uptake beyond the ilioinguinal nodes on pedal lymphoscintigraphy. Thus, although considered technical failures, this may in fact be a true finding. It appears possible that those with no uptake on pedal lymphoscintigraphy may be unlikely to show uptake with DCMRL, although numbers are too small to make definitive conclusions. Of the 9 patients in whom contrast uptake was absent on one side, this may be due to an absence, functional aplasia or hypoplasia of the lymphatic system, technical failure to access the lymph nodes, or needle displacement. Hypoplastic or absent lymphatics has been shown to be useful for confirming or completing the clinical diagnosis in conditions with primary lymphatic anomalies.^30^

#### Dynamic aspect of DCMRL allows for more detailed study of flow in the lymphatics and therapy compared to noncontrast MR imaging and lymphoscintigraphy

**Figure 5:**
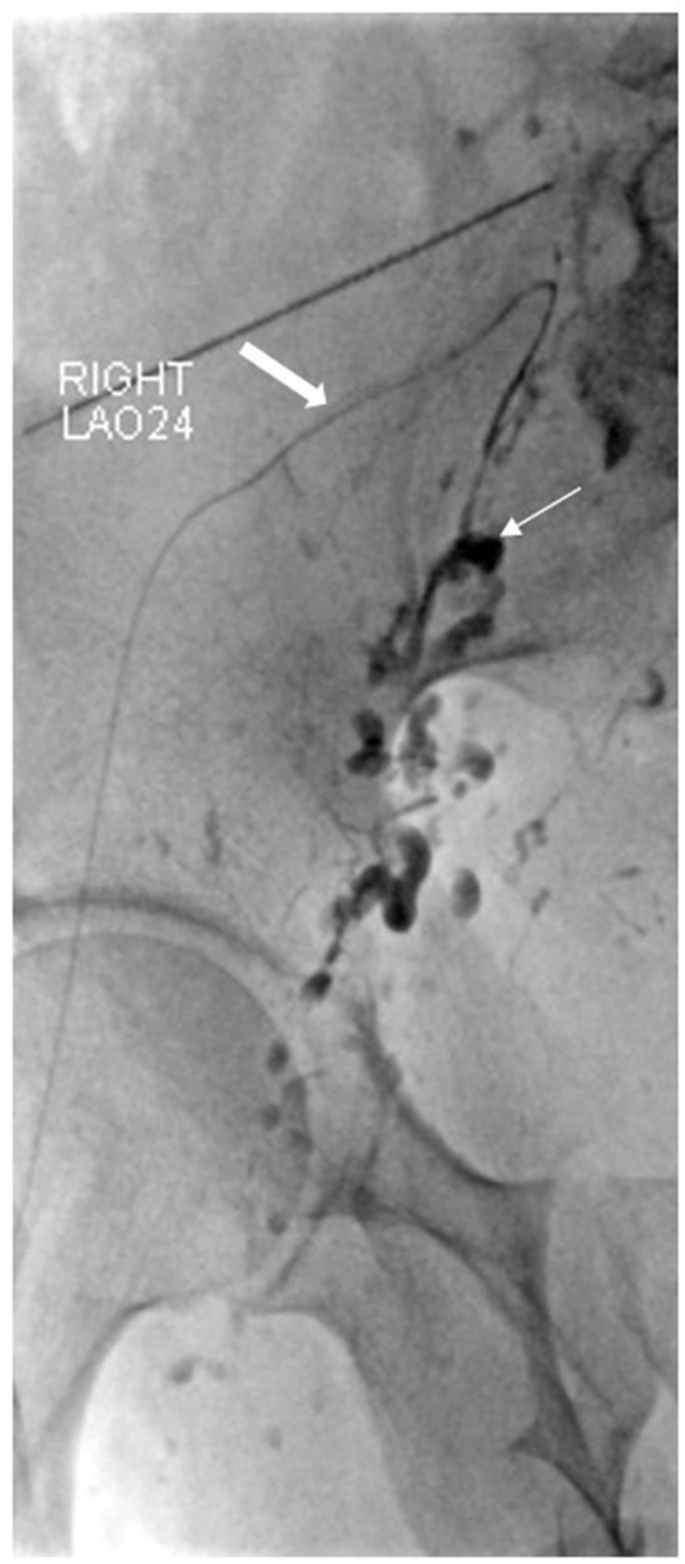
Glue embolization carried out on a patient with reflux (Case 28 from Fig 2). Lipiodol lymphangiogram first carried out with injection from left groin lymph node. Refluxing channels targeted fluoroscopically and glue embolization (thin white arrow shows glue cast in embolized lymphatics) carried out after microcatheter (white arrow) catheterization of the lymphatic vessels.

For example, 3 of the patients found to have reflux into the contralateral side was not known about prior to DCMRL. Two of these patients subsequently underwent glue embolization [Fig 5] with improvement in the number of lymphatic blisters and volume of chylous leak, and no further episodes of infection on the affected side in both patients.^31^

**Figure 6:**
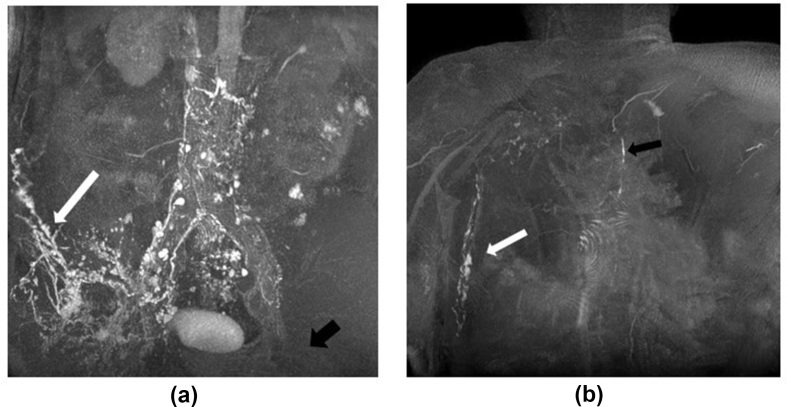
A 66-year-old male patient (Case 28) with right leg and genital lymphedema. Previous surgery with removal of right groin lymph nodes thus only the left was injected. Postcontrast maximum-intensity projection (MIP) shows contrast injected from left groin node (black arrow) has refluxed to the right; then rerouting was done around the right flank (white arrow shows refluxed contrast starting to track up right flank) (6a). Delayed imaging showed the contrast from the right flank tracking up the chest wall (white arrow) before draining via further collaterals into the TD (black arrow) (6b). Images shown were acquired with the Dixon technique. TD = thoracic duct.

Five patients demonstrated superficial rerouting of the lymphatics confirming obstruction of their normal lymphatic pathways [Fig 6 and Table 2]. Confirmation of central lymphatic obstruction can then lead to exploration of surgical options such as lymphovenous anastomosis.^32^^,^^33^

An inherent difficulty arising from investigating rare diseases is the relatively small numbers of patients with each condition; however, the imaging has led to improved understanding of the mechanisms behind lymphatic anomalies and changes in patient management. The development of the technique has resulted in refinements over time, which has made the data more heterogenous. Our experience is presented here to facilitate the development of DCMRL services elsewhere within the UK.

## Conclusion

DCMRL has proven to be a safe technique for imaging the central lymphatic anatomy of patients with primary lymphatic anomalies without any adverse effects that were observed with lipiodol lymphangiography. Increased uptake of this imaging modality will be invaluable in the phenotyping, classification, and management of patients with primary lymphatic anomalies.

## Funding

1. This paper has been supported by a joint grant from the Medical Research Council (MRC) and the British Heart Foundation (BHF) (MR/P011543/1 and RG/17/7/33217), with no direct involvement of these sponsors.
2. The authors declare that they have no conflict of interest.

## Author contribution

1 guarantor of integrity of the entire study: Ratnam.
2 study concepts and design: Ratnam, Mills, Wale, Itkin, Howe.
3 literature research. Ratnam, Wale, Mills, Howroyd.
4 clinical studies: Ratnam, Howroyd, Mills.
5 experimental studies/data analysis: Ratnam, Mills, Wale, Howroyd.
6 statistical analysis: Ratnam.
7 manuscript preparation: Ratnam, Mills, Wale, Howroyd.
8 manuscript editing: Ratnam, Mills, Wale, Howroyd, Howe, Gordon, Mansour, Ostergaard, Mortimer.
9 funding acquisition: Ostergaard, Mortimer and Mansour

## Conflict of interest

The authors declare the following financial interests/personal relationships which may be considered as potential competing interests: L A Ratnam reports financial support was provided by UKRI Medical Research Council. If there are other authors, they declare that they have no known competing financial interests or personal relationships that could have appeared to influence the work reported in this paper.
